# AngoraPy: A Python toolkit for modeling anthropomorphic goal-driven sensorimotor systems

**DOI:** 10.3389/fninf.2023.1223687

**Published:** 2023-12-22

**Authors:** Tonio Weidler, Rainer Goebel, Mario Senden

**Affiliations:** ^1^Department of Cognitive Neuroscience, Faculty of Psychology and Neuroscience, Maastricht University, Maastricht, Netherlands; ^2^Maastricht Brain Imaging Centre, Faculty of Psychology and Neuroscience, Maastricht University, Maastricht, Netherlands

**Keywords:** goal-driven modeling, computational modeling, deep learning, reinforcement learning, sensorimotor control, cortex, anthropomorphic robotics, recurrent convolutional neural networks

## Abstract

Goal-driven deep learning increasingly supplements classical modeling approaches in computational neuroscience. The strength of deep neural networks as models of the brain lies in their ability to autonomously learn the connectivity required to solve complex and ecologically valid tasks, obviating the need for hand-engineered or hypothesis-driven connectivity patterns. Consequently, goal-driven models can generate hypotheses about the neurocomputations underlying cortical processing that are grounded in macro- and mesoscopic anatomical properties of the network's biological counterpart. Whereas, goal-driven modeling is already becoming prevalent in the neuroscience of perception, its application to the sensorimotor domain is currently hampered by the complexity of the methods required to train models comprising the closed sensation-action loop. This paper describes *AngoraPy*, a Python library that mitigates this obstacle by providing researchers with the tools necessary to train complex recurrent convolutional neural networks that model the human sensorimotor system. To make the technical details of this toolkit more approachable, an illustrative example that trains a recurrent toy model on in-hand object manipulation accompanies the theoretical remarks. An extensive benchmark on various classical, 3D robotic, and anthropomorphic control tasks demonstrates AngoraPy's general applicability to a wide range of tasks. Together with its ability to adaptively handle custom architectures, the flexibility of this toolkit demonstrates its power for goal-driven sensorimotor modeling.

## 1 Introduction

Goal-driven modeling is a novel approach in computational neuroscience that utilizes deep learning to construct highly performant models of brain function (Yamins and DiCarlo, 2016). By relying on computational optimization, this approach obviates the need for hypothesis-driven construction and hand engineering of brain models. Instead, such models emerge when deep neural networks with an appropriate architecture (e.g., layers resembling interareal pathways and topography), appropriate activation functions, and biologically meaningful input modalities are trained on natural stimuli and ecologically valid tasks. The resulting models can be probed *in silico* to yield hypotheses about yet-undiscovered computational mechanisms in the targeted structures. These, in turn, can then be tested *in vivo*. The enormous potential of this approach has been attested by recent successes within the neuroscience of perception (Yamins and DiCarlo, 2016). In particular, convolutional neural networks (CNN) have been shown to be effective models of the visual (e.g., Yamins et al., 2014; Kubilius et al., 2018; Schrimpf et al., 2020) and auditory (e.g., Kell et al., 2018; Li et al., 2022) cortex. Linear neural response prediction (Carandini et al., 2005; Cadieu et al., 2007; Yamins et al., 2014; Yamins and DiCarlo, 2016), representational similarity analysis (Kriegeskorte and Douglas, 2018), or composite metrics like brain-score (Schrimpf et al., 2020) have repeatedly validated learned representations and their hierarchy as at least reminiscent of representations in the brain.

However, at present, the advantages of goal-driven deep learning are limited to the study of perception and do not translate well to the study of the sensorimotor system. At the same time, this domain would have much to gain from the goal-driven approach. The limitations of hand-engineering already apparent when modeling open-loop systems such as sensory cortices become even more prominent for the closed perception-action loop: The complexity of the sensorimotor system renders the construction of a complete computational account by hand untenable (Loeb and Tsianos, 2015). Moreover, reductionist models limiting themselves to an isolated phenomenon neglect the idea of emergence (Ellis, 2008) which postulates that unique properties or behaviors of a system also arise from the *interactions between* its components. Yet, hardly any research has so far been conducted in the direction of goal-driven sensorimotor models. This is because goal-driven deep learning is typically based on data-intensive supervised learning. While datasets required to optimize models to perform perceptual tasks are widely available, those necessary to optimize models on sensorimotor tasks are sparse. Michaels et al. (2020) collected recordings from tracking gloves worn by monkeys during a grasping task and trained a recurrent neural network (RNN) on latent representations of simulated visual input to predict recorded muscle velocities. The goal-driven model reproduced the neural dynamics recorded in a neural interface in the monkeys' AIP, F5, and M1. However, reproducing muscle velocities is a much narrower task than generating overt behavior. Accordingly, mapping specific input stimuli to specific muscle (or joint) activations limited to an individual's grasping strategies might not actually represent an ecologically valid task setting. Additionally, gathering such recordings *in vivo* is costly and time-consuming. A better solution would avoid the need for labeled data. Reinforcement learning (RL) presents the (currently) most potent solution of that sort. Here, an artificial agent (e.g., a brain model) learns to adopt some behavior by autonomously exploring a task environment and adapting its decision making to maximize an external reward that indicates the task objective. Recent advances in deep learning-based robotic control (OpenAI et al., 2019, 2020; Huang et al., 2021) demonstrate the ability of this approach to teach RNNs complex, ecologically-valid tasks such as in-hand object manipulation *in silico*. Unfortunately, training such systems requires substantially more computation and tuning than classification tasks, and applying RL algorithms to recurrent convolutional neural networks (RCNN, where *convolutional* usually describes the network's ability to process visual information) is a nontrivial engineering effort. Therefore, we believe that goal-driven sensorimotor control research would dramatically benefit from a flexible but easy-to-use toolkit for training complex biologically constrained neural networks in an efficient RL setup.

To address this, we introduce *AngoraPy* (**An**thropomorphic **Go**al-directed **R**esponsive **A**gents in **Py**thon), a Python library for the efficient, distributed training of large-scale RCNNs on anthropomorphic robotic tasks with human sensory input modalities. AngoraPy is explicitly tailored toward the use of goal-driven deep learning within computational neuroscience. It bridges the technical gap between modeling deep neural network representations of the brain and training them on complex tasks at scale. It thereby facilitates the use of goal-driven deep learning for sensorimotor research by requiring no profound knowledge about the underlying machine learning techniques from its user. Every aspect of the modeling process is highly customizable. AngoraPy can train any Keras (Chollet, 2015) model adhering to domain-specific constraints on inputs (humanoid sensation) and outputs (motor commands). Tasks, including their objectives and reward function, as well as anthropomorphic body models, are entirely customizable in a native API built on a strong backbone of state-of-the-art physical simulation software (Todorov et al., 2012) and community-favored standards for task interfaces (Brockman et al., 2016; Towers et al., 2023).

In the following, we first give a brief overview of modeling the sensorimotor system and reinforcement learning (Section 2). Subsequently, Section 3 describes the framework in detail along with a practical example. AngoraPy aims to provide a flexible tool that is agnostic to task and model definitions. To showcase this flexibility, we present both the results of the practical example and a battery of benchmarks that demonstrate out-of-the-box performance on diverse task sets and networks (Section 4). We then conclude by summarizing our work and discussing prospects for future applications (Section 5).

## 2 Background

Research on sensorimotor control investigates the mapping of sensory stimuli to motor commands constituted by the coupling between sensory cortices and motor cortex. Taking into account the effect of motor action on sensory stimuli (both directly and indirectly), this coupling establishes a closed-loop feedback control system. The study of its controller, the nervous system, often starts with a hypothesis embedded in or abstracted from a model. For instance, trajectory control was an influential model of movements under perturbation but was later shown to be incomplete when the goal is not the trajectory itself, but a static target (Cluff and Scott, 2015). Models of metabolic muscular energy consumption often base their cost functions on the heat and power observed in individual or groups of muscles (Tsianos et al., 2012). Other work uses kinematic measurements and electromyography data to select the most suitable among various candidate minimization criteria (Pedotti et al., 1978). All these models begin with a hypothesis about a mechanism sourced inductively from behavioral or physiological data.

Another line of research is concerned with the functional roles of the different cortical areas involved in sensorimotor control. To this end, decoding studies using, e.g., functional magnetic resonance imaging (fMRI) have successfully identified neural representations related to movement parameters. For example, Mizuguchi et al. (2014) have shown that right frontoparietal activity reflects the intended force level in grasping. Using multi-voxel pattern analysis in fMRI data, Gallivan et al. (2013) found activity in frontoparietal regions that predicts the movements of the participating limbs and the actions of the hand during grasping. Similarly, Filimon et al. (2015) discovered that the voxels that encode action-related information during reaching movements spread over vast portions of both the premotor and posterior parietal regions. These lines of work started with a hypothesis (or several) about a candidate role for some region, pathway, or network. However, research that simultaneously elucidates the operation of large sensorimotor networks and the contribution of individual regions remains scarce (Gallivan et al., 2018).

Although valuable in their own right, none of the current approaches can provide a comprehensive perspective on the sensorimotor system. First, hand-engineered models scale poorly to more intricate processes involving larger cortical networks. Second, from the perspective of Marr's three levels of analysis (Marr, 1982), these models only touch on the first two levels of explanation. The implementation of the mechanisms (i.e., the third level) in terms of neural circuits and biological constraints still requires substantial work (Franklin and Wolpert, 2011). This is also and maybe particularly true regarding the study of neural transformations, rendering even the second level only partially covered. While decoding studies provide valuable information about the information encoded in various regions, they cannot provide insights into the neurocomputational strategies by which these representations are transformed within and across brain regions. Due to the complexity of these transformations, it is also nearly impossible to hand-engineer them. Similarly, it is unlikely that fitting deep models to reproduce recorded neural responses in multiple areas would yield great success given that data for such an approach are lacking (Yamins and DiCarlo, 2016).

### 2.1 Reinforcement learning-based goal-driven modeling

Reinforcement learning-based goal-driven modeling overcomes these issues. Navigating the complexity of the neurocomputations to be modeled is handled by optimization, and training data is generated autonomously on-the-fly. Reinforcement learning (RL) optimizes models to approximate a mapping π(*s*_*t*_) → *a*_*t*_ from state descriptions *s*_*t*_∈*S* to actions *a*_*t*_∈*A* that maximizes the sum of rewards *r*_*t*_ allocated to a trajectory (episode) of state-action pairs. In contrast to supervised and unsupervised learning, RL does not rely on pre-collected data but on self-generated samples gathered by acting in the environment. The mapping π(*s*_*t*_) constitutes a (behavioral) policy parameterizable by any function approximator, but in the setting described here, the policy is implemented by a deep neural network that models (parts of) the brain. Physically (albeit in simulation), the agent is situated in its environment such that its actions affect the environment's progression through time.

**Figure 1:**
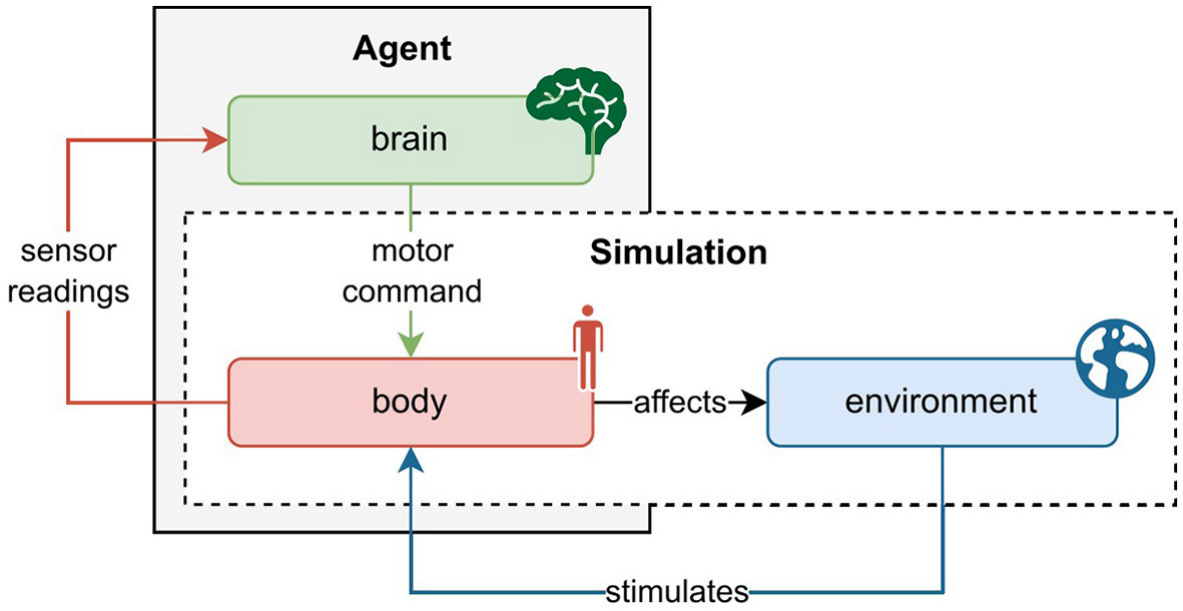
Schematic interaction between agent and physics simulation. The agent consists of a brain and a body, whereas the physics simulation covers the environment and body. As such, the agent's body constitutes the interface between the brain and the environment. At every time step in the simulation, the environment causes an effect on the body which generates sensory stimulation. The body's sensors read this stimulation and communicate the information to the agent's brain. The brain then maps the description of the perceived state to a motor command which it sends back to the body. The body executes the action and thereby affects the environment as well as itself. With readings of the new sensory state, this cycle recurs until the environment ends the episode, a trajectory of state transitions.

Figure 1 depicts the relation between agent and simulation, and the interaction of *body, brain*, and the *environment*. Generally, this interaction follows the following schema. The environment triggers sensory stimulation in the agent's body, which then feedbacks the vectorized perception of that stimulation to the agent's brain. The brain maps the sensory information to a desired motor command and projects it to the body. The body executes the motor command and thereby affects the environment. The new state of the environment produces the next sensory stimulation of the body, so the circle closes. Alongside describing the state, the environment also rewards the action performed in the previous state. On the basis of this information, the brain's parameters can be optimized.

### 2.2 Plausibility, validation, and hypothesis generation

Notably, the process by which learning occurs in this setup need not be biologically plausible. It suffices that biological and artificial agents have approximately the same objective, defined by some reward-based reinforcement signal. This is an innate characteristic of any RL algorithm. Freeing the training procedure from biological constraints allows highly complex tasks to be learned in a feasible time using state-of-the-art machine learning techniques. We describe AngoraPy's implementation of these techniques in Section 3.

Trained models can be validated with the help of, e.g., decoding studies or representational similarity analysis (Kriegeskorte et al., 2008). From validated models reproducing real cortical representations, hypotheses about, e.g., transformations can be extracted. These can then be tested by making predictions for new data on how some signal develops throughout the cortex. Therefore, goal-driven deep reinforcement learning complements hypothesis-driven research on the sensorimotor system as a method of *hypothesis generation*.

Interestingly, hypotheses can also be generated when the computational model and experimental data do *not* fit (Loeb and Tsianos, 2015). Conditions under which such errors occur can inspire empirical research to identify relevant biological or physical constraints whose inclusion may refine the goal-driven model. *In silico* ablation studies on these constraints then shed light on their potential functional roles.

## 3 Method

In this section, we will provide an overview of the design of AngoraPy, which trains models that combine an anthropomorphic motor plant with a deep neural network. We begin by outlining our core design principles, followed by an introduction to the framework's main dependencies. Next, we provide a technical overview of AngoraPy's key components, including a prototypical workflow that illustrates the process of building a model of in-hand object manipulation. It is not our aim to hereby present a definitive model, but to demonstrate how researchers can utilize AngoraPy to define and train their own models.

Table 1 summarizes deployment options for AngoraPy and their intended usage. **AngoraPy is open source and available on GitHub**^1^ and licensed under GNU GPL-3.0. The repository also gives installation instructions for the different deployment options. In addition to the Python API, command-line scripts are available as entry points with extensive customization options. The software is tested and maintained under Linux, but the Python API is not specific to an operating system by design. We also maintain a growing collection of hands-on tutorials^2^ and documentation.

**Table 1:** Deployment options available for AngoraPy.

	**Description**	**Intended usage**
Source	The open-source code is available on GitHub. On the repository, a master branch corresponds to the latest release version and the development branch features more recent changes and additions.	Contribution, Development versions
PyPI	All release versions are available on the Python Packaging Index (PyPI) and can thus be easily installed via pip.	Local installation
Docker	A Dockerfile is available on GitHub and can serve as is for a clean installation of AngoraPy with all its dependencies, GPU support, and MPI support. This also allows for easy deployment on HPC clusters.	Local installation, HPC installation

Additional details and installation instructions are available on GitHub.^1^

### 3.1 Principles and goals of design

We have built AngoraPy with appropriate design principles in mind: *Neuroscience First, Modularity*, and *Pragmatism*. These guide the implementation toward the overall goal of providing a flexible but effective tool to neuroscientists, and draw inspiration from the principles used to build PyTorch (Paszke et al., 2019).

As we put **neuroscience first**, AngoraPy by design addresses the needs of neuroscientists who want to build goal-driven models with ease. It is our main objective to provide an intuitive API that interfaces background processes without requiring elaborate knowledge about them. This entails limiting options to only those that truly benefit neuroscientific research. AngoraPy does not aim to be a comprehensive reinforcement learning or deep learning library. In the remainder of this section, we will motivate these choices and options.

AngoraPy is built for **modularity**, where options matter. The API is general enough to support a wide range of applications. In particular, AngoraPy is not specific to tasks or models. However, to guarantee easy interaction with the framework, both need to follow requirements (we lay them out in Sections 3.3 and 3.6). As development continues, these requirements are monitored and adjusted when needed.

Lastly, the framework is built with **pragmatism**. Computational efficiency is a matter of high importance in AngoraPy. At the same time, performance matters, and we aim to provide tools that achieve state-of-the-art results. However, neither efficiency nor performance should substantially hinder the other, nor should the simplicity of the API suffer from either. As such, we try to maintain a somewhat Pareto-optimal balance between the three objectives.

### 3.2 Main dependencies

Before detailing the framework itself, it is useful to discuss the choice of underlying libraries that provide its backbone. This choice is not arbitrary, as it has implications for the modeling conducted by *users* of AngoraPy. The rise of deep learning as a toolbox of algorithms in many fields was greatly accelerated by the increasing availability of well-designed, easy-to-use, and flexible autodifferentiation packages. Since the library introduced here trains deep neural networks as models of the brain, it also builds on such software. The two most prominent packages currently available are *PyTorch* (Paszke et al., 2019) and *TensorFlow/Keras* (Chollet, 2015; Abadi et al., 2016). However, *JAX* (Bradbury et al., 2018) is also becoming popular among fundamental researchers in deep learning due to its low-level flexibility. Choosing among these options is not always obvious since each of them has sufficient functionality to support any research. In AngoraPy, our aim was to cater not only to the contributors' needs and expertise, but, of course, also to that of the end user. This is relevant since we neither aim nor consider it useful to hide the implementation of model architectures behind a native API. Instead, models are built using the API provided by the autodifferentiation library. AngoraPy targets neuroscientists at an arbitrary level of deep learning experience, not researchers in machine learning. Selecting the API AngoraPy relies on for model design thus focuses on popularity, accessibility, and robustness, and not particularly on the needs of machine learning researchers.

By choosing Keras as the modeling API supported by AngoraPy, we decided on a long-established option that matches the experience of many researchers and provides an easy modeling interface with high flexibility. The persistent popularity of Keras on StackOverflow, Kaggle, PyPi, and Google Colab (Team Keras, 2021) is a testament to its popularity with a general audience. Furthermore, Keras and Tensorflow are considered more accessible to beginners who are not yet familiar with any such library, making them a good fit for neuroscientists newly exploring goal-driven deep learning. Lastly, Keras is currently under intense redesign to remove its exclusive dependence on TensorFlow and reimagine it as a *backend-agnostic*, top-level API that interfaces TensorFlow, JAX, *and* PyTorch. In conclusion, Keras as a model design backend in AngoraPy not only adequately reflects current end-user demands but also presents a safe choice for future shifts within the deep learning community.

With Keras, AngoraPy covers the *brain* component of the agent. To cover *body* and environment, we use a native MuJoCo+Gym(nasium) stack. MuJoCo (Todorov et al., 2012) is a recently open-sourced physics simulator offering high computational accuracy and speed. Gym (Brockman et al., 2016) and its successor, Gymnasium (Towers et al., 2023), offer an interface for easy communication with an environment in reinforcement learning applications. For AngoraPy, we have built a native wrapper for Gym(nasium) environments, extending their functionality to suit the needs in an anthropomorphic setting better and to simulate body parts and their interaction with the world in MuJoCo.

### 3.3 Tasks and simulation

In goal-driven modeling, the task definition is crucial to the methodology's premises. It sets the *goal* and thereby *drives* learning to generate the neurocomputations one seeks to discover. However, prescribing appropriate task and simulation constraints is not trivial.

AngoraPy ships an API specifically designed for anthropomorphic tasks. It extends the Gym(nasium) library (Brockman et al., 2016; Towers et al., 2023) by interfaces and wrappers that inject additional features and standards tailored toward sensorimotor applications. This comprises adapted interfaces to the official Python bindings of MuJoCo (Todorov et al., 2012) and for implementing anthropomorphic task environments. Several built-in dexterity tasks employing a simulation of an anthropomorphic robotic hand (the Shadow Dexterous Hand^3^) exemplify such implementations and can serve as a foundation for dexterity research. They are implemented on top of a hand model and task implementation originally included but discontinued in Gym. Beyond these built-in tasks, the task API can be used to define custom tasks and reward functions that concern either existing or new, custom body parts.

**Example**. *In the current and the following example segments, we will illustrate the construction of a goal-driven in-hand object manipulation model in AngoraPy using a toy architecture. To this end, example segments annotate the upcoming technical descriptions with practical illustrations. In-hand object manipulation (IHOM) is a manual dexterity task. To simulate it, we use the hand model shipped with AngoraPy. It consists of* 24 *joints and has its palm connected to a fixed socket via a joint with two degrees of freedom. Actuators are directly attached to the joints and apply control in terms of the absolute desired joint angles. Of the* 24 *joints, four are coupled. Thus, they cannot be controlled directly but move dependent on other joints. Accordingly, the motor plant has a total of* 20 *degrees of freedom. In-hand object manipulation covers a broad category of tasks, but teaching it to an artificial agent requires a prototypical specification. Consistent with OpenAI et al. (**2020**), we prototype the manipulation task as the in-hand reorientation of a cube whose faces are uniquely colored and labeled. A target reorientation is specified as an angle of rotation around a fixed point (the object's center) and is achieved if the cube's rotation angle lies within* η *units of the target angle; that is, their distance* *d*_*g*_(*t*) ≤ η*. To encourage stable behavior toward the end of a reorientation, we define a single episode as a chain of reorientations. Thus, the agent needs to learn manipulation in a manner that maintains sufficient control to enable it to perform the next reorientation from the endpoint of the previous. Per reorientation (i.e., goal), the agent is given* 8 *seconds and the total number of possible reorientations is capped at* 50*. The* 8*s-timer resets for every goal reached. The episode ends immediately when the cube is dropped, indicated by the cube center's* *z* *position coming below that of the palm*.

When AngoraPy instantiates a task environment, it encapsulates it with a wrapper that connects external (e.g. native Gym) environments with its own API. Specifically, state descriptions are converted to *Sensations*, the canonical input type for AngoraPy, which we will introduce in Section 3.4. Optionally, the wrapper accepts preprocessor modules that transform observed states and rewards. AngoraPy offers two built-in preprocessors that normalize rewards and states, respectively, via running mean estimators. Such transformations can be crucial in many tasks (Ilyas et al., 2020) but hamper progress in others. For instance, reward normalization is not useful with environments that assign constant rewards for nonterminal states. As the number of steps per episode approaches infinity, the reward for nonterminal states approaches zero, while that for terminal states approaches (negative) infinity. As episodes last longer, the relative importance of the terminal state thus increases, potentially destabilizing learning.

A key component of an RL environment is, furthermore, the reward function. In AngoraPy, reward functions are integrated into environments as the union of a *reward prototype function* and a *reward configuration*. This setup enables parametrization and inheritance, and thereby facilitates objective-related ablation experiments. Additionally, nonstatic reward functions allow for shifts in the balance between different terms in the reward function. These shifts can create training schedules that facilitate learning.

**Example**. *To model the IHOM objective through a reward function, we provide the same feedback as OpenAI et al. (**2020**) on success (r*_s_*), failure (r*_f_*), and progress (r*_p_*)*,


(1) $$\begin{array}{l} r_{task}\left(t\right)=r_{p}\left(t\right)+5I_{s}\left(t\right)-20I_{f}\left(t\right)-0.1 \end{array}$$

*The indicator variables* *I*_s_ *and* *I*_f_ *are equal to* 1 *in success and failure (dropping), respectively (and* 0 *otherwise). Progress* *r*_p_(*t*) *at time* *t* *is defined as*


(2) $$\begin{array}{l} r_{p}\left(t\right)=d_{g}\left(t-1\right)-d_{g}\left(t\right), \end{array}$$

*where* *d*_g_(*t*) *is the distance between the current rotation of the cube and the orientation of the goal at time* *t. The progress term* *r*_p_(*t*) *also captures regression away from the goal. During training, a reward preprocessor estimates the running mean and variance of the reward function to normalize its output*.

### 3.4 Sensation

To learn the task, the agent must find a favorable mapping between states and actions. AngoraPy trains custom biologically inspired networks to control anthropomorphic, simulated bodies. Accordingly, the state *s*_*t*_ on which the policy π(*s*_*t*_) conditions its action distribution needs to emulate human sensory modalities. At present, these modalities comprise *vision, touch*, and *proprioception*, as they are key components of human sensorimotor processing (Wolpert et al., 2011). Although the task goal could also be implicitly encoded in either of these, states additionally feature an explicit *goal* description. One may consider these explicit goal representations internal representations, as encoded in, e.g., areas of the prefrontal cortex (Miller and Cohen, 2001; Braver, 2012). Together, this 4-tuple constitutes the (vectorized) input to the model, contained within the *Sensation* type. Keeping the tuple's elements separate allows for multiinput architectures, as required when modeling sensory modalities that exclusively target their respective sensory cortices.

**Example**. *For manipulation, all three sensory modalities are relevant feedback. However, to simplify the problem for the purpose of this example, we omit vision and replace it with immediate information on the object's pose. Additionally, the model requires a goal description. The environment returns this as a vector representation of the target quaternion. A preprocessor (as described in Section 3.3) records the means and variances across all modalities and uses these to normalize the states*.

#### 3.4.1 Auxiliary data for asymmetric value functions

Importantly, goal-directed modeling aims at plausible inference. As discussed in Section 2, the learning procedure itself, however, need not be biologically plausible for its premises to hold. During training, this distinction can be leveraged. First, this concerns the learning algorithm that can be applied. Second, we can enrich the state with auxiliary information only used during training (Pinto et al., 2018). The training algorithm used by AngoraPy requires a value network $V^{\pi }\left(s_{t}\right)$ that estimates the expected value of being in state *s*_*t*_ given the current policy π. Since *V*^π^ is discarded at inference time, it can rely on any biologically implausible, *auxiliary* input without harming the model's plausibility.

**Example**. *We provide auxiliary information to the value network during training. These include fingertip positions, the relative orientation between the target and the current object orientation, the object's positional velocity, and the object's rotational velocity. Together with sensory and goal inputs, these constitute the Sensation instances produced by the manipulation environment. AngoraPy automatically feeds these to the relevant parts of the model*.

### 3.5 Motor commands

An agent's brain maps its sensations to motor commands. AngoraPy trains stochastic policies, and thus maps to the parameters of probability distributions. The executed motor commands are directly sampled from that distribution. Like its human counterpart, an anthropomorphic agent has continuous control over multiple action dimensions. This entails the approximation of a *joint* probability density function (PDF). One assumes the joint distribution over possible actions to be i.i.d. marginal distributions. In practice, modeling joint PDFs can become problematic because the space of potential actions is infinite. With a growing number of marginal distributions, this makes predictions about interactions difficult. Therefore, it proves beneficial to bin each continuous action variable in applications with many degrees of freedom (DoF; OpenAI et al., 2019, 2020). This converts the problem to the approximation of a multicategorical distribution.

**Figure 2:**
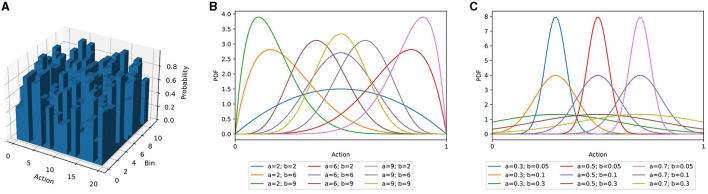
Three different policy distribution classes as described in Section 3.5. **(A)** Multicategorical distribution. **(B)** Beta distribution for different combinations of α- and β-values. **(C)** Gaussian distributions with different means and standard deviations. For equal α and β parameters, the Beta distribution resembles the Gaussian distribution. For diverging parameters, it becomes increasingly skewed. Importantly, the Beta distribution's domain is entirely confined to the interval [0, 1].

AngoraPy offers different options for modeling the probability distribution of a multiple-DoF continuous policy: the multicategorical (binned), Gaussian, and Beta distributions and an interface for implementing custom policy distributions. In the following sections, the three built-in options are outlined alongside their (dis)advantages. Figure 2 depicts their characteristic shapes.

#### 3.5.1 Multicategorical control for continuous action spaces

In the multicategorical setting, a continuous action space *A* consisting of *n* actions *a*∈[*a*_*min*_, *a*_*max*_] is segmented into *b* bins per action, each spanning $\frac{1}{b}\left(a_{max}-a_{min}\right)$ units of the continuous space. Accordingly, the model has an output space *A*∈ℝ^*b*×*n*^. Compared to a continuous policy distribution, this tends to be substantially larger. However, the problem is simplified for two reasons: First, binning the action space leads to a finite set of possible actions that the robot can execute. In contrast, a theoretically infinite number of actions can be sampled from the continuous distribution. Second, over a finite set of possible actions, optimization has full control. A multicategorical distribution can have any shape imaginable, while Gaussian and Beta distributions have characteristic shapes (Figure 2).

#### 3.5.2 Gaussian-distributed continuous control

Gaussian policy distributions predict the means and standard deviations of every marginal distribution. The output space of an *n*-DoF control task is thus 2*n*-dimensional. Standard deviations are usually better trained independently from the input. That is, the standard deviations are themselves a set of directly optimized parameters, but the input state is irrelevant to their manifestation in the policy output. Essentially, the optimization then slowly anneals the standard deviations to 0 as it becomes more confident in predicting the means. Predicting the standard deviation as a function of the input is possible but less stable when facing outliers in the state space. It also adds additional load to the optimization because it requires approximating an evolving mapping between instances and confidence.

#### 3.5.3 Beta-distributed continuous control

In bounded action spaces, the infinite support of Gaussian distributed policies biases them toward the limits (Chou et al., 2017). The unconstrained support requires a fold of all predictions outside of an action's bounds onto the boundary values and artificially increases their probability. Chou et al. (2017) demonstrated that an agent predicting the parameters of a Beta distribution circumvents this issue. The support of the Beta distribution is in the interval [0, 1], independent of the parameters, and is parameterized by α and β (Figure 2). By scaling samples to the allowed interval of the action, the prediction is complete, but bias-free. Chou et al. (2017) showcased the positive effect on several RL algorithms (Schulman et al., 2015; Wang et al., 2017), and we also found this to apply to the algorithm used in AngoraPy (detailed in Section 3.7.1; see Section 4.2 for a demonstration of the Beta distributions effectiveness). In motor control, actions are constrained by maximum joint flexion and extension. The Beta distribution hence is a natural choice for many anthropomorphic systems.

**Example**. *To learn in-hand object manipulation, we train the agent with a multicategorical distribution. The model then predicts a vector* *a*∈ℝ^*db*^ *where* *d* *is the degrees of freedom of the motor plant (*20 *for the ShadowHand) and* *b* *is the number of bins per degree of freedom. Consistent with OpenAI et al. (**2020**), we set* *b* = 11.

### 3.6 Brain models

**Figure 3:**
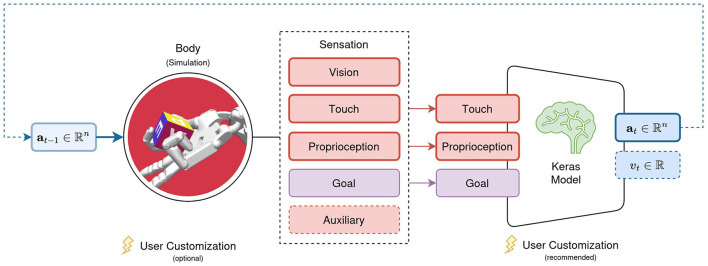
Requirements for models trained with AngoraPy. The set of input modalities must be a (sub)set of the modalities available in a Sensation. Outputs must be a policy head output following the desired distribution's shape, followed by a value output head projecting to a single scalar.

With **s**_*t*_ and **a**_*t*_ in π(**s**_*t*_|θ) → **a**_*t*_ covered in the previous sections, the following focuses on the neural network models parameterized by θ. We argued in Section 3.2 for Keras as the backbone of their implementation. AngoraPy can train any model implemented in Keras if it adheres to two constraints. First, the input layers of the model must be a subset of the modalities present in a *Sensation*. Second, the model must be available as a tuple of policy, value, and joint networks, where joint is the combination of policy and value networks into a single network with two heads. The policy network's output must be built by the agent's policy distribution, and the value network must map the input onto a single scalar. These requirements are depicted in Figure 3.

Often, a deep model of the sensorimotor system will require a recurrent convolutional neural network (RCNN) implementation. Recurrence affords memory and representations of environmental dynamics. Convolutional networks mimic the hierarchical and retinotopic structure of the visual cortex (LeCun et al., 2015). Furthermore, their layers learn representations that resemble those found in the respective cortical areas (LeCun et al., 2015; Yamins and DiCarlo, 2016). During training, AngoraPy employs several strategies to deal with the computational load of RCNNs during training (Section 3.7). However, hardware will always constrain the viable complexity of a model.

**Example**. *The toy model that we use in this example is a shallow but high-dimensional recurrent neural network with an embedding layer and implements the architecture used before by OpenAI et al. (**2020**). Different modalities from the input Sensations we defined previously will first be concatenated into a single vector and then fed into the embedding layer. This toy model architecture is available as a built-in baseline in AngoraPy's network registry (under the key wide). Recurrence is necessary to model the dynamics of the motion of external objects because neither rotational nor positional velocities are directly available in the raw sensory state space*.

#### 3.6.1 Weight sharing between policy and value networks

Because at train time, optimization relies on state value estimates, networks need additional *value heads*. These are trained to predict the average future reward received under the current policy when in the given state. As we explained in our discussion of asymmetric value functions (Section 3.4), the value head can be discarded at inference time. Its architecture, therefore, needs not be biologically plausible. However, when designing a network architecture, incorporating the value head plays an important role. Essentially, one must decide whether to entirely separate the value from the policy network or at what stage policy and value heads diverge. Separation can lead to better peak performance since optimization need not balance between two objectives. However, since value estimation and action selection will overlap in the information they need to extract from the input, sharing the stem of their networks can lead to faster convergence. Naturally, auxiliary inputs can only be integrated where heads diverge.

**Example**. *We build the toy model without a shared base to prioritize performance over convergence time. The policy network and the value network have the same architecture up until after the recurrent layer. The policy network culminates in a distribution head generated by the policy distribution. The value head projects to a single linear scalar predictive of the value of the state described by the given input*.

#### 3.6.2 Modularity and pretraining

Sensorimotor systems are vast and include various cortices. Generally, this tends to make deep implementations of sensorimotor systems inherently modular. For instance, subnetworks that model sensory cortices are generally interchangeable. Extensive research on goal-driven models in the sensory domain allows modelers to easily exploit this modularity. First, researchers investigating specific cortical areas in the context of sensorimotor processing can enrich their model by integrating it with other models. On the other hand, different models of the same brain structure can be plugged into existing sensorimotor models to evaluate their effect on functional performance and neurocomputational validity. Finally, upstream models like sensory cortices can be pretrained on functions identified in perception research. This severely disburdens the RL training on the main task. Naturally, it might also prove beneficial for the validity of downstream neurocomputations if upstream feature extraction is bootstrapped with less task specificity.

### 3.7 Training agents

Before, we described building models and tasks and their interaction as communicated by sensations and motor commands. This sets the rules for the core of AngoraPy, the distributed deep reinforcement learning backend. AngoraPy employs proximal policy optimization (PPO; Schulman et al., 2017) to train its agents' policies using stochastic gradient descent (specifically Adam; Kingma and Ba, 2014). PPO is an RL algorithm that alternates between data *gathering* and *optimizing* in continuous cycles. During the gathering phase, experience is collected by acting in the environment following the current policy. During the optimization phase, this experience is used to update the policy. This asynchronicity between data generation and optimization allows for distributed, sample-efficient training, as Section 3.7.3 will detail.

#### 3.7.1 Proximal policy optimization

Proximal policy optimization (Schulman et al., 2017) directly optimizes the selection of actions. This avoids the proxy of evaluating every action in a given state. Gradients are calculated with respect to the parameterized policy π_θ_(**a**_*t*_|**s**_*t*_). In essence, the objective tries to move the policy in a direction that increases the likelihood of beneficial actions and decreases that of disadvantageous ones, captured by advantage estimate Â(**a**_*t*_, **s**_*t*_)∈ℝ. However, traversing the policy space in this way might apply steps too large to permit stable learning. PPO addresses this issue by scaling the update by the ratio *r*_*t*_(θ) between the currently optimized π_θ_(**a**_*t*_|**s**_*t*_) and the previous version of the policy at gathering time π__θ_*old*___(**a**_*t*_|**s**_*t*_). By clipping this ratio, potentially excessive updates are avoided. This is implemented in the following objective:


(3) $$\begin{array}{l} L{\left(\theta \right)}^{PG}=-\min\left(r_{t}\left(\theta \right)Â\left({\text{a}}_{t},{\text{s}}_{t}\right),\text{clip}\left(r_{t}\left(\theta \right),1-\epsilon ,1+\epsilon \right)Â\left({\text{a}}_{t},{\text{s}}_{t}\right)\right) \end{array}$$

Here, $r_{t}\left(\theta \right)=e^{\pi_{\theta }\left({\text{a}}_{t}|{\text{s}}_{t}\right)-\pi_{{\;}_{\theta_{old}}}\left({\text{a}}_{t}|{\text{s}}_{t}\right)}$ is the logarithmic ratio. The minimum in Equation (3) removes the lower bound if the advantage is positive (Schulman et al., 2017). If the advantage is negative, we want to decrease the ratio. The clipping bounds by how much we want to decrease it. The minimum ensures that recoveries from deteriorated policies receive gradients (instead of being clipped away).

The advantage *Â*(**a**_*t*_, **s**_*t*_) is the difference between the return collected from taking action **a**_*t*_ and the average future return ${\bar{R}}_{t}$ from being in state **s**_*t*_, estimated by the value network *V*_θ_(**s**_*t*_) with a mean squared error loss


(4) $$\begin{array}{l} L{\left(\theta \right)}^{V}={\left(V_{\theta }\left({\text{s}}_{t}\right)-R_{t}\right)}^{2}. \end{array}$$

The *R*_*t*_ that serve as a target to *V*_θ_(**s**_*t*_) come from the same samples the policy network learns from. Thus, *V*_θ_(**s**_*t*_) boils down to a state-conditioned running mean estimator. The estimator may be a separate network or share part of its parameters with the policy network. In any case, it is convenient (and required for the latter) to implement the optimization of both *V*_θ_(**s**_*t*_) and π_θ_(**a**_*t*_|**s**_*t*_) jointly, hence combining their objectives into one joint cost function


(5) $$\begin{array}{l} J\left(\theta \right)=L{\left(\theta \right)}^{PG}-c_{v}L{\left(\theta \right)}^{V}+c_{e}H\left(\pi_{\theta }\right), \end{array}$$

where *c*_*v*_ can be adjusted to prevent *J*(θ)^*V*^ from dominating *J*(θ)^*PG*^ when sharing parameters between value and policy network. *H*(π_θ_) is the entropy of random variable π_θ_. A stochastic policy's entropy can be seen as a measure of the degree to which the agent still explores. Thus, incorporating it (scaled by constant *c*_*e*_) into the objective discourages premature convergence (Williams and Peng, 1991; Williams, 1992).

**Example**. *Since we previously made the decision not to share parameters between policy and value network, setting* *c*_*v*_ *is unnecessary. The clipping range is kept at* ϵ = 0.2*, the value recommended by Schulman et al. (**2017**). We include an entropy bonus. In the literature, the entropy coefficient* *c*_*e*_ *is often in the range* [0, 0.01] *(Mnih et al.*, *2016**; Schulman et al.*, *2017**; Engstrom et al.*, *2020**; OpenAI et al.*, *2020**). We set* *c*_*e*_ = 0.001 *at a fairly low value because the entropy of independent marginal distributions is the sum over marginal entropies, which naturally increases the entropy bonus*.

#### 3.7.2 Truncated backpropagation through time

During training, long input sequences to recurrent layers can overload memory. That is because backpropagation through time (BPTT) needs to record the activations throughout the entire sequence to calculate gradients. Truncated BPTT (TBPTT; Williams and Peng, 1990) tackles this issue by dividing the sequence into subparts, over which the gradients are backpropagated. At the end of each subsequence, the RNN's state(s) are passed over to the next subsequence, but the computation graph is cut off. This alleviates the need for memory in sacrifice for well-modeled long-distance dependencies (LDD) and precise gradients. However, in practice, LDDs are often negligible if we hypothesize the recurrence to encode external dynamics mostly. Imprecise gradients have likewise proven to be of limited harm. AngoraPy thus natively uses TBPTT to balance precision and memory efficiency. Note that TBPTT is often compulsory where episodes are long and networks are large. However, where this is not the case, TBPTT can be turned off by setting *s* to the length of the episode.

**Figure 4:**
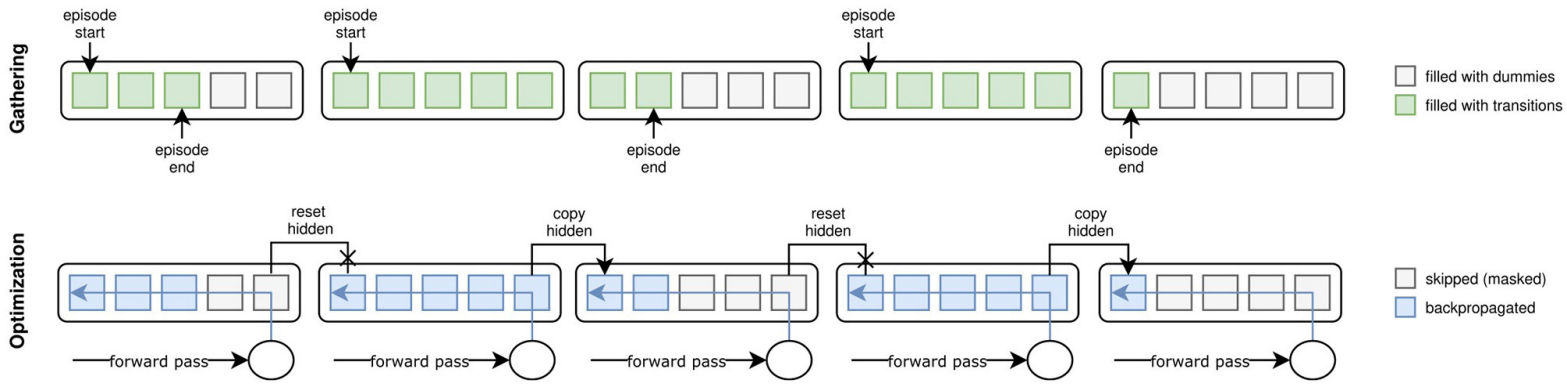
Truncated Backpropagation Through Time (TBPTT) during gathering **(top)** and optimization **(bottom)**. During gathering, the transition sequence is cut off every *k* elements. If the episode ends before a cutoff point, the remainder of positions up to the next cutoff points is filled with dummy transitions. During optimization, this leads to a dataset of equally sized sequences. Dummy transitions are masked when backpropagating the error. Cutoff points at nonterminal states are handled by copying the last hidden state of the previous sequence into the initial hidden state of the current one.

During the gathering phase, we collect data points in subsequences of length *s* if the policy is recurrent. Each worker initializes a buffer with all-zero matrices **B**∈ℝ^(*h*/*s*) × *s*×*n*^ for each collected transition information where *n* is the length of the vector representing the piece of information. The buffer matrices are progressively filled with the transitions recorded while stepping through the environment. Whenever a subsequence of length *s* is filled, it is pushed to the buffer, and the next subsequence is started. If an episode finishes before the end of a subsequence, its current state is pushed to the beginning of the buffer's subsequence, and the worker skips *s*−(*t* mod *s*)−1 time steps (where *t* is the current time step). Given the buffer initialization, this essentially fills the remainder of the sequence with zeros. During optimization, these zeros are masked to be ignored when calculating gradients. Tuning *s* attempts to fit the number of previous time steps relevant to modeling the dynamics of the environment. Thus, it is task-dependent. Figure 4 visualizes the implementation of TBPTT in AngoraPy.

**Figure 5:**
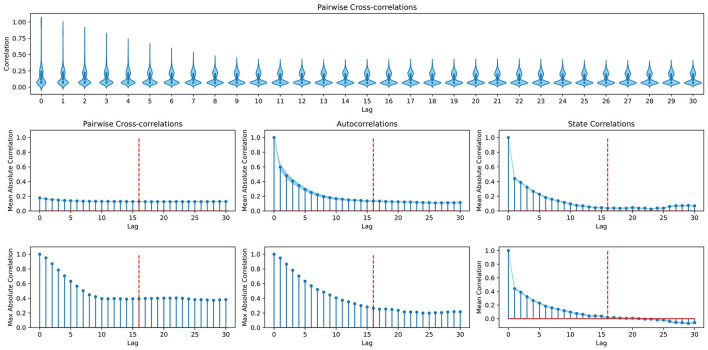
Analysis of the dynamics in the in-hand object manipulation environment to determine the temporal dependencies between state variables. **(Top row)** Violin plots depicting the distribution of cross-correlations between singular variables at each lag. **(First column)** Mean and maximum cross-correlations between singular variables at different lags. **(Second column)** Mean and maximum autocorrelations (each variable is only compared to itself) at different lags. **(Third column)** Correlations between state vectors (as opposed to time series of single variables) taken at different lag distances. Dashed red lines mark lag 16, at which the here presented example cuts off gradient propagation to past time steps. All plots are based on 15 i.i.d. time series of 100 time steps that were collected by randomly taking actions in the environment. Standard errors around the mean are indicated by light blue shaded areas.

**Example**. *To capture the dynamics of IHOM, we set* *s* = 16 *which equates to* 1.28 *s. This covers the approximation of external velocities by relating spatial to temporal distances between two consecutive time steps. As an additional means of determining a reasonable* *s, one can consult an analysis of the temporal dependencies of states in the environment. Consider* *Figure 5**, where we plot different perspectives on auto- and cross-correlations between steps at different lag displacements. Although looking at the maximum cross- and autocorrelations reveals dependencies beyond a lag of* 16*, it is also evident that the strongest dependencies tend to lie within lag* 16 *and thus we set* *s* = 16.

#### 3.7.3 Distributed computation

At the core of AngoraPy's ability to train large-scale models on complex tasks lies its native distributed design. Learning in a recurrent, convolutional setting can require extensive training on a large amount of data. To manage this during simulation and optimization, high-performance computing on a distributed system is inevitable. Proximal policy optimization provides the algorithmic foundation necessary for AngoraPy to implement this. As PPO generates data asynchronously, any number of parallel gathering workers (GW; usually allocated to a thread/core of a CPU) can simulate the environment and roll out the policy independently. Similarly, optimization can be spread over multiple optimization workers (OW; preferentially GPU enabled) by calculating and merging gradients on partial minibatches.

**Figure 6:**
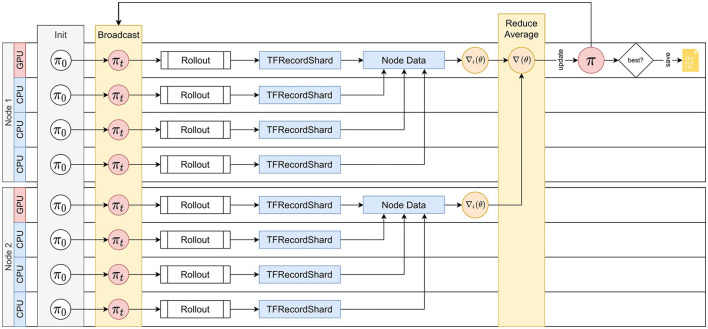
Distributed Training with MPI. An exemplary depiction of the distributed training cycle comprising gathering and optimization on two compute nodes. Distribution scales to an arbitrary number of nodes. Spawned processes (e.g., on every CPU thread or core) all share the load of gathering information. The GPU(s) on a node is assigned to the lowest-rank process of the node, and all GPU processes share the computational load of optimization. At cycle 0, every process initializes the policy, which is then synced to match the single initial policy on the root process. Every process then rolls out the policy to generate experience up to horizon *T*. The data is stored in shards. Every optimization process then collects and merges the shards produced by workers on the same node. Based on this data, the optimization process calculates a gradient. The gradients of all optimizers are then reduced into one by averaging and applied as an update to the policy on the root process. π_new_ is then broadcasted to all processes, which repeat the cycle by rolling out the new policy.

AngoraPy implements this bipartite distribution strategy under the Message Passing Interface (MPI). The detailed process is depicted in Figure 6. MPI spawns multiple processes running the same script, each building a full copy of the agent. At initiation, virtual workers are evenly allocated to processes if possible. Optimally, the allocation is bijective. Forcing uneven allocation by creating a number of virtual workers not divisible by the number of available CPU workers would substantially waste computational time. During the gathering phase, every worker acts as a GW. Each GW rolls out the policy and generates a trajectory of experience up to horizon *T*. The worker then temporarily saves this trajectory on disk. During the optimization phase, the data is evenly assigned to all OWs. Every OW reads its data share from the disk and calculates a partial gradient. In between batches, the partial gradients are accumulated among all OWs, and the total gradient is applied to the policy weights.

Note that while complex tasks involving recurrent and convolutional networks may require distributed computation, many simpler tasks such as finger oppositions or anthropomorphic robots with less degrees of freedom (e.g., locomotion) do not. AngoraPy can flexibly be applied without, with little (e.g., on local workstations or laptops) or extensive (on supercomputing clusters) parallelization. On systems without GPU support, AngoraPy instead distributes the optimization amongst CPU processes. Distributed computation is thus purely a feature, not a constraint.

In the given example segments, we describe an application that scales the distribution capabilities of AngoraPy to the capacity of large high-performance computing (HPC) clusters. However, such resource-rich computing environments are not necessary to make productive use of either AngoraPy itself or its distribution strategies. Local workstations suffice to train policies sufficiently complex to be of ecological relevance, as verified by the benchmarks described in Section 4.2. All of these can be trained locally on a consumer-grade workstation, including the anthropomorphic dexterous task of reaching.

##### Batch tiling

The combination of distributed optimization and the division of sequences into chunks that need to remain in order requires batches to be tiled. Tile shapes are defined by the number of trajectories *n* and chunks *m* per trajectory where *nm* must equal the number of chunks per update. Balancing between the two presents a tradeoff between temporal bias and trajectory bias. The more chunks included per trajectory, the lower the bias of the update toward a specific temporal segment of the trajectories. At the same time, the resultant lower number of independent trajectories will inevitably produce a bias toward specific trajectories. However, particularly during early training, independent trajectories differ vastly in their course. Thus, temporal links between trajectories disappear early, and temporal biases dissolve. When AngoraPy automatically determines tile shapes based on the number of chunks per update and the number of available optimizers, it thus finds the valid shape with the highest number of trajectories included at the same time. To support this process, it is generally a good practice to choose combinations of batch and chunk sizes that produce chunks per update divisible by the number of available optimizers.

**Example**. *Data collection is distributed among* 384 *CPU workers, and we use* 32 *GPUs for optimization. Every gathering worker generates* 2, 048 *time steps. We use a batch size of* 12, 288 *transitions. As TBPTT groups transitions into chunks of length* 16*, a total of* 768 *chunks are divided among optimizers. Every cycle entails* 64 *updates to the current policy each epoch and* 192 *updates per cycle. The batch size is intentionally high to lower the number of updates per cycle. Too many updates would cause the policy to move away from the data-generating policy. Additional updates would thus increasingly optimize based on off-policy data and conflict with the on-policy assumption of PPO's objective function*.

### 3.8 Saving agents, resuming experiments, and monitoring

Training automatically stores the model's best-performing parameters between cycles, if the model's performance during the gathering phase surpasses the previous best performance. Additionally, AngoraPy always saves the newest version of the model. Optionally, the training procedure can save the model's state in intervals, for instance to later probe the model at different levels of progress. Model storing uses Keras' built-in formats, but the parameters of the Adam optimizer and those needed to reload the agent object (mostly hyperparameters) are captured separately in NumPy and JSON files. This strategy allows agents to be loaded both for evaluation purposes *and* to resume training.

A *Monitor* can be connected to the training process to additionally log the training process. The monitor tracks rewards, episode lengths, losses, and preprocessor statistics and logs hyperparameters. Additionally, it records several statistics about training, including runtimes and memory usage. All log files are in JSON format and thus are both human- and machine-readable. The progression traces are live updated during training and can be conveniently monitored during or after training using a native web application offering a filterable and searchable overview of all experiments stored on the machine and graphs visualizing stored progression traces and statistics for specific experiments or comparing multiple.

## 4 Results

In this section, we present the results of exemplary experiments to demonstrate the efficacy of AngoraPy's RL backend. Specifically, we demonstrate its ability to handle different task categories and model architectures. The experiments were designed to highlight the versatility and robustness of the toolkit and include benchmarks on various tasks, from classical control to robotics. The outcomes of these experiments provide compelling evidence of the effectiveness of AngoraPy in developing performant goal-driven anthropomorphic models of any shape and purpose.

### 4.1 In-hand object manipulation with a toy model

**Figure 7:**
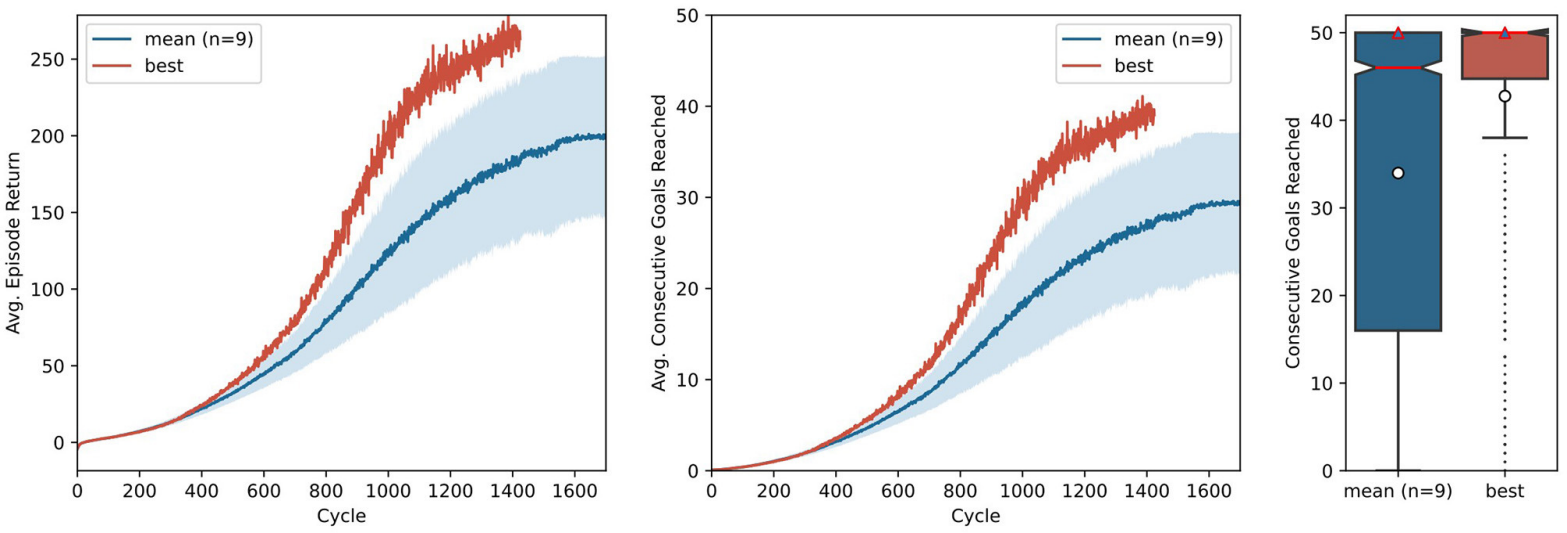
Training of in-hand object manipulation agents using a toy model. Average episode returns **(left)** and average goals achieved per episode **(middle)** show the progression of performance over training cycles. The mean performance over 9 independent runs is shown in blue, with a shaded 95% confidence interval. The curve of the best performing agent is shown in red. The distribution (boxplot) of 480 episodes over the number of the consecutive goals reached **(right)** shows the performance of the agent after training. Red lines indicate the median, white circles indicate means, and red triangles modes.

To render the technical description of the key components of AngoraPy more intelligible, we provided a concrete example of applying the full framework using the model suggested by OpenAI et al. (2020). In the context of the present work, it acts as a simple toy model without any biological plausibility and serves to demonstrate AngoraPy's efficacy in training complex anthropomorphic tasks. The results for this example are depicted in Figure 7. Agents reliably converge toward a strategy chaining an average of 30 goals. Convergence occurs after ~1, 500 cycles, corresponding to 96 h of training in our setup employing 384 CPU cores (Intel Xeon E5-2690 v3) for data collection and 32 GPUs (NVIDIA Tesla P100 16 GB) for optimization.^4^ At this point, the agent has seen 1, 187, 512, 320 samples, equating 3.012 years of experience. The best performing agent converges at a similar speed, but chains ~40 goals.

Note that Figure 7 shows two metrics of performance: The cumulative reward based on the task set in the example segment in Section 3.3, and the number of consecutive goals reached (CGR) within one episode. Although the former differs from the latter by incorporating the distance between initial position and goal, the two metrics provide nearly identical insights. Nevertheless, when communicating the performance of goal-driven models trained by RL, we recommend to report interpretable auxiliary metrics (like CGR for IHOM), instead of cumulative rewards. This abstracts away the actual performance from the specific reward function used and even allows one to compare their variants. Most importantly, however, it enables readers who are less familiar with reinforcement learning terminology or who may not be acquainted with the specific reward function to quickly assess the power of a model.

After training, we evaluated the best performing version of the toy model (i.e., the set of weights that achieves the maximum average results in its gathering cycle) on 480 independent episodes. Here, actions are no longer sampled from the policy distribution, but instead the most probable action is chosen deterministically. The distribution of these episodes as measured by CGR is shown in Figure 7. It can be observed that while on average the agents chain ~34 reorientations, in most episodes they actually chain the maximum possible 50 reorientations, as discerned from the mode. The best agent is highly stable, with both the median and the mode at 50.

Our results show that while most agents reliably converge to a reasonable performance (30 CGR or higher), outliers can occur. Specifically, both time-to-convergence and peak performance vary between runs. Designing biologically plausible models can mitigate this variance. By constraining the flow of information and its integration, the policy space can be transformed to favor specific solutions to the problem. At the same time, since policies are similar in their manipulation strategy but only differ in the effectiveness of their execution, the observed variance between agents can also be interpreted as an interindividual difference in a neuroscientific context.

The architecture we employed in this example was proposed in the seminal work of OpenAI et al. (2020). It is *not* biologically plausible but serves as a good baseline for assessing our tool. The training performance that we report in Figure 7 differs from OpenAI et al. (2020) mainly because the network relies only on raw sensory information and because batch sizes and the amount of data gathered per cycle differ for practical, hardware-related reasons.

### 4.2 Benchmarks

AngoraPy aims to provide out-of-the-box functionality on a wide range of tasks. The following section presents benchmarking experiments on classical and sensorimotor tasks implemented by Gym (Brockman et al., 2016) to demonstrate the general applicability of AngoraPy to various problems beyond the one showcased above. Naturally, this set of tasks is not exhaustive. Nevertheless, the following indicates the general-purpose applicability of the stack of methods underlying AngoraPy's training framework.

#### 4.2.1 Classical control

**Figure 8:**
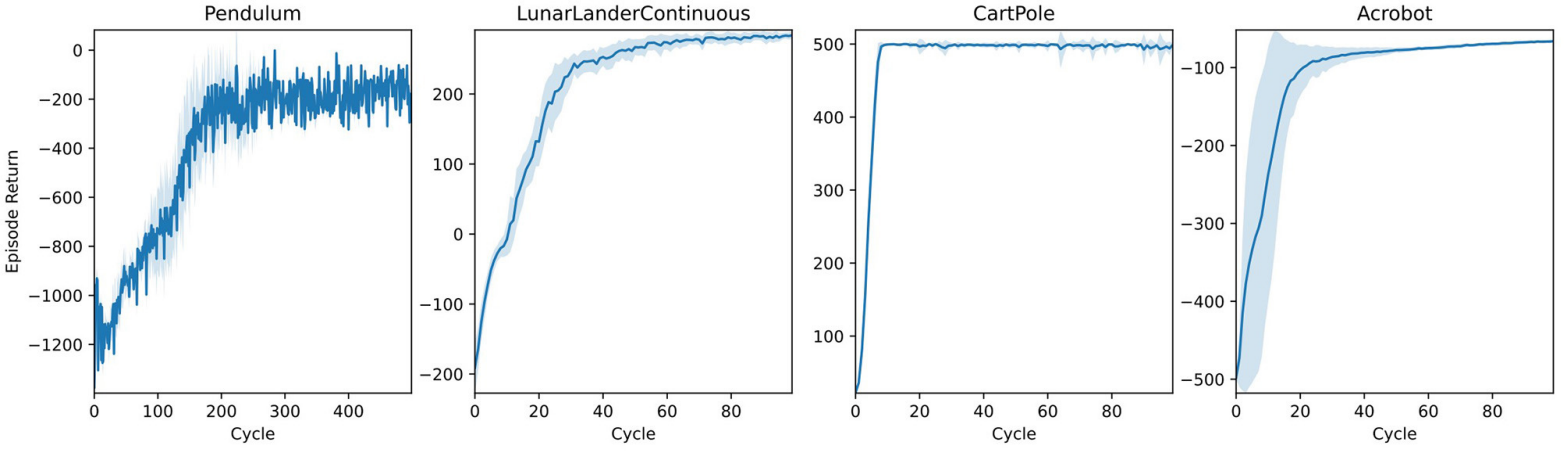
Learning curves of agents trained on different classical control tasks implemented by Gym (Brockman et al., 2016). All agents use the same two-layer neural network architecture with a Beta/categorical policy head. Weights were not shared between policy and value network. Blue lines represent the average returns of 16 independent agents, and the lighter area around them is their standard deviation.

Pendulum equilibration tasks constitute a standard benchmark for control systems. We present learning trajectories for the Gym implementation of three variants in Figure 8 alongside a classic trajectory optimization for a 2D spacecraft (LunarLander). Without any specific parameter tuning, AngoraPy's PPO implementation solves all tasks.

#### 4.2.2 3D control in MuJoCo

**Figure 9:**
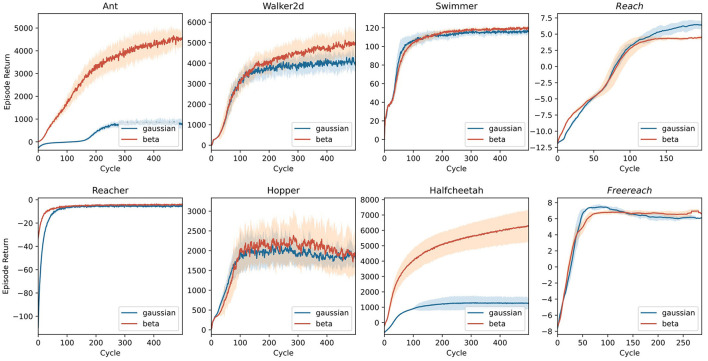
Benchmark experiments on three-dimensional control tasks simulated in MuJoCo (Todorov et al., 2012) and implemented by Gym (Brockman et al., 2016). Tasks with *italic* titles are anthropomorphic reaching tasks. In all other tasks, the model controls non-anthropomorphic motor plants. The latter agents use the same two-layer architecture used in Figure 8. For anthropomorphic tasks, a recurrent network with an LSTM cell builds the policy. Lines represent the average returns of 16 independent agents, and the lighter area around them is their standard deviation. Blue lines correspond to agents using a Gaussian policy, whereas red lines summarize the performance of Beta distributed policies.

Whereas the control tasks in Figure 8 are easy to solve for most state-of-the-art RL algorithms, the set of (robotic) control tasks benchmarked in the first three columns of Figure 9 pose a more significant challenge. This challenge stems (i) from a more rigid simulation in three-dimensional space, (ii) from purely continuous control, and (iii) in some cases, from higher degrees of freedom. Nevertheless, PPO again demonstrates an impressive invariance in its applicability to different robots using the same parameters. Note that for most of the environments tackled in Figure 9, the original paper introducing PPO (Schulman et al., 2017) already presented benchmarks, yielding similar results with differences stemming from different parameter settings. Agents using Beta distributions often outperform or are on par with their Gaussian counterparts on these tasks.

#### 4.2.3 20-DoF dexterous control

One of the most fascinating and complex problems in the motor domain is sensation-guided manual control. The complexity of the hand as a motor plant stems from high degrees of freedom coupled with nontrivial dependencies between joints. To solve any task involving this plant, the model first needs to learn the relationship between control applied to the joints and its effect on the position of the plant's individual parts. A task focusing on this raw skill thus is a good benchmark and initial proof-of-concept for any model of the sensorimotor system. In Figure 9, the last column shows results for two manual dexterity tasks, *reaching* and *freereaching*. Both tasks challenge the agent to join the thumb's fingertip with one other digit's fingertip (target finger) as indicated by the goal description. In *reaching*, we model this task by rewarding the proximity of individual fingertips to locations sampled around their initial position for nontarget fingertips and sampled around a meetup point for the thumb's and the target's fingertip. In *freereaching*, we abstract away from predetermined locations and instead formulate the reward as the inverse distance between the thumb and the target finger, and add a punishment for other fingertips entering a zone around the thumb's fingertip. As demonstrated in Figure 9, both formulations of the task can be reliably learned within 100–150 cycles.

## 5 Discussion

The present article introduces *AngoraPy*, a Python library that allows researchers to build goal-driven models of the human sensorimotor system at scale. This library specifically targets computational neuroscientists and aims to empower the community by offering a tool that simplifies the setup and training of large-scale neuroconnectionist sensorimotor models of high functional validity. AngoraPy provides a distributable reinforcement learning (RL) backend that is accessible through a high-level, easily comprehensible API. Thereby, it allows its users to train large recurrent convolutional neural networks (RCNNs) as models of the brain without the need for precollected data, and features standards for defining model architectures and tasks. AngoraPy's task API is implemented on top of Gym(nasium) (Brockman et al., 2016; Towers et al., 2023), and the constituent bodies and environments of the tasks are simulated in MuJoCo (Todorov et al., 2012). A synergy of wrappers and interfaces standardizes the configuration of anthropomorphic task environments. These ship together with predefined environments for dexterity tasks, including variants of finger tapping (reaching) and in-hand object manipulation. Available sensory modalities comprise vision, touch, and proprioception, or a subset thereof. Motor commands are issued by stochastic policies and are sampled from multivariate distributions over the joint space of the motor plant. The construction of models is standardized but flexible. AngoraPy supports multimodality, weight sharing, asymmetric policies, and pretrainable components. The reinforcement learning backend implements proximal policy optimization (Schulman et al., 2017) and embeds it in a stack of supportive methods. To support large RCNNs applied to long-lasting tasks, the gradient-based optimization chunks sequences and truncates out-of-chunk time steps. Both simulation and optimization are highly scalable through native MPI distribution strategies. Taken together, AngoraPy combines the power of state-of-the-art reinforcement learning with high-performance computing in a sensorimotor modeling toolbox.

We demonstrated AngoraPy's capability to train arbitrary network architectures on several task domains, including anthropomorphic robots with up to 20 degrees of freedom. Our benchmarking results highlight that no extensive hyperparameter tuning is necessary to successfully apply AngoraPy to new tasks, which makes the toolbox particularly suitable for users who do not have experience with reinforcement learning. The wide range of robotic motor plants used in these benchmarks, from one-legged bodies (*Hopper*) to bipedal walkers (*Walker2d*) to five-finger hands (e.g., *Reaching*), furthermore shows that the toolkit's applicability is by no means confined to a specific part of the body.

In an example that accompanied the technical details of the presented software, we illustrated the workflow of applying AngoraPy to study anthropomorphic motor functions in more detail. This example did not aim to present a novel model, but rather to demonstrate the functionality of AngoraPy. As such, we trained a simple toy model without biological plausibility but adhering to the anthropomorphic input and output constraints posed by AngoraPy's modeling standards. The toy model acts as a placeholder for carefully designed model architectures developed by the research that the tool presented here seeks to empower. Such research conducts extensive surveys of biological data and aggregates them to develop novel inductive biases that can be implemented in the network model.

As inductive biases become increasingly constraining, the range of possibly emerging neurocomputational strategies should be reducing. Given that these biases adequately reflect the anatomical and biological constraints of the brain, the neuroconnectivity shaping thereunder will better fit the solutions employed by the brain. The careful, data-driven design of these biases is thus not only the most eminent, but also a substantial work. By publishing AngoraPy, we intend to empower researchers to focus their work on this intricate part of modeling and alleviate the effort of integrating and training their brain models as part of the sensorimotor loop.

Previously, there had been a lack of software that thoroughly supports goal-driven modeling in the sensorimotor domain. Existing libraries that offer high-quality implementations of various RL algorithms (e.g., Guadarrama et al., 2018; Moritz et al., 2018; Raffin et al., 2021) are primarily intended for use by RL researchers. This is reflected on the one hand in the wide range of covered algorithms, which comes at the cost of user-friendliness for non-experts. Additionally, as they are nonspecific in nature, general RL libraries often require substantial engineering efforts when applied to training anthropomorphic sensorimotor models. Moreover, they forfeit out-of-the-box flexibility with respect to applications in favor of flexibility with respect to algorithms. As a consequence, applying an algorithm to a specific problem can become cumbersome if the flexible standards of the library do not align well with the requirements of the problem. With AngoraPy, we instead offer the research community a comprehensive tool specifically tailored to its application in neuroscience. In neuroscientific modeling, where the choice of the RL algorithm is less important, such a domain-specific tool is more efficient and easier to use and, therefore, better suited than general RL libraries.

AngoraPy does not, in principle, limit the level to which users can customize brain, body, and task models. Nevertheless, more extensive customizations will require more effort from the user to implement. AngoraPy enables this without necessitating direct modification of the library by making most of its components available to the end user. Only the training algorithm itself is not available for customization because training, unlike network, body, and task, is not part of the biologically inspired model. Instead, it is a tool to build the model. Importantly, we do not require users to make all possible customizations, as we offer thoughtfully selected default parameters, procedures, and built-in models and tasks. However, *some* components must be customized by the end-user to generate *novel* outcomes and models.

Although AngoraPy is highly flexible in its support for brain model architectures, certain network layouts may still conflict with its training procedure. We currently explicitly account for fully connected, convolutional, feedforward, and recurrent networks, and any combination thereof. As we continue to actively expand AngoraPy, we monitor trends in the goal-driven modeling community and adapt AngoraPy accordingly.

With its comprehensive set of features, AngoraPy can be used by neuroscientists who seek to build models of sensorimotor systems that bridge across Marr's (1982) levels of description of neural information processing. Specifically, our framework addresses the algorithmic implementation of a sensorimotor function guided by both computational theory and biological constraints. Observing under which constraints biologically valid organization emerges (and under which it does not) may additionally support our understanding of the interplay between structure and function. The resulting goal-driven models can then be utilized to evaluate existing hypotheses, as well as to generate novel predictions to guide theoretical and empirical research. For example, AngoraPy may be used to develop models of the frontoparietal network with biologically inspired pathways and regions trained on in-hand object manipulation tasks to better understand the neurocomputations that underlie human dexterity. These models may then generate new hypotheses about information processing that occurs within and between areas in both feedforward and feedback directions. Similarly, AngoraPy may be employed to study complex motor skills such as grasping, reaching, manipulating, balancing, or locomotion.

## Data availability statement

Software introduced in this paper is available at: https://github.com/ccnmaastricht/angorapy and licensed under GNU GPL-3.0.

## Author contributions

TW developed the software introduced in the manuscript. TW wrote the first draft of the manuscript. TW and MS contributed to manuscript revision. MS and RG secured funding for the project. TW, MS, and RG read and approved the submitted version.

## Footnotes

^1^ https://github.com/ccnmaastricht/angorapy

^2^ https://github.com/weidler/angorapy-tutorials

^3^ https://www.shadowrobot.com/dexterous-hand-series/

^4^The number of GPUs in this setup is a byproduct of how the HPC architecture that we use is set up and should not be taken as a guideline. Usually, a substantially lower number of GPU optimizers should suffice.
